# Lower Lean Mass Is Associated with Greater Arterial Stiffness in Patients with Lower Extremity Artery Disease

**DOI:** 10.3390/jpm11090911

**Published:** 2021-09-13

**Authors:** Viktoria Muster, Katharina Gütl, Gudrun Pregartner, Andrea Berghold, Leyla Schweiger, Philipp Jud, Marianne Brodmann, Gerald Seinost

**Affiliations:** 1 Division of Vascular Medicine, Department of Internal Medicine, Medical University of Graz, 8036 Graz, Austria; katharina.guetl@medunigraz.at (K.G.); leyla.schweiger@medunigraz.at (L.S.); philipp.jud@medunigraz.at (P.J.); marianne.brodmann@medunigraz.at (M.B.); gerald.seinost@medunigraz.at (G.S.); 2 Department of Medical Informatics, Statistics and Documentation to Institute for Medical Informatics, Statistics and Documentation, Medical University of Graz, 8036 Graz, Austria; gudrun.pregartner@medunigraz.at (G.P.); andrea.berghold@medunigraz.at (A.B.)

**Keywords:** arterial stiffness, lower extremity artery disease, body composition, lean mass

## Abstract

Background: Arterial stiffness is independently associated with lower extremity artery disease (LEAD). Although obesity is already known as an independent cardiovascular risk factor, it was found that, paradoxically, in patients diagnosed with cardiovascular disease, an increase in body mass index (BMI) was associated with a decrease in mortality. However, the underlying mechanism of this paradoxical association remain uncertain. In this study, we firstly hypothesize that arterial stiffness correlates with body mass; secondly, the underlying mechanism of the association for patients with LEAD is individual body composition, in particular, lean mass. Methods: The present study was performed as a single-center, prospective, observational analysis. A total of 412 patients with current or previously diagnosed LEAD (Rutherford Classification 2–4) were included, the cfPWV and AIx were measured as indices of arterial stiffness, and a body composition assessment was performed. Results: In male patients, there was a significantly negative correlation between the AIx and lean mass coefficient (*p* = 0.004, 95% CI: −0.28 (−0.48–0.09)). Conclusion: For patients with peripheral arterial disease, our data show that lower lean mass in male patients is associated with increased arterial stiffness as measured by the AIx. Therefore, progressive resistance training may be beneficial for the reduction in arterial stiffness in PAD patients in secondary prevention.

## 1. Introduction

Numerous studies have shown that arterial stiffness, measured by the carotid-femoral pulse wave velocity (cfPWV) or aortic PWV, as well as the augmentation index (AIx), is associated with a higher cardiovascular disease (CVD) risk and event rate for a first or recurrent major CVD event in high-risk groups and in the general population. It is also independently associated with lower extremity artery disease (LEAD) [1,2,3,4], as well as atherosclerosis and diabetes [5,6]. More recent studies found that an elevated pulse pressure in combination with a reduced total systemic compliance are independently associated with cardiovascular events. This finding suggests that arterial stiffness, in addition to an increased pulse pressure, is a risk factor for future vascular events [7,8,9]. Moreover, in the Baltimore Longitudinal Study on Aging (BLSA), it was found that the cfPWV increases with age in men as well as in women. Structural changes in the vascular media such as elastin content reduction, increased collagen, elastin fractures, and calcification have been linked to an increased cfPWV [10]. Nevertheless, it was also found that vascular stiffening increases with age in populations with minor or no atherosclerosis, indicating that in an aging population, vascular stiffening occurs independently of atherosclerosis [11]. The CVD burden may be determined by an increased arterial stiffness, especially in older people [12]. Several studies found that the cardiovascular system is affected by the aging process, including structural changes of the heart and vessels, as mentioned above, as well as an increase in arterial stiffness [10,13,14,15,16]. Data from observational studies additionally showed that normotensive individuals with an increased arterial stiffness develop hypertension more often [10].

Although obesity is already known to be an independent cardiovascular risk factor [17,18,19], it was found that in patients with known CVD, an increasing body mass index (BMI) was paradoxically associated with a decrease in mortality [20,21,22,23], but no such data are available for patients with LEAD. However, the underlying mechanism of this paradoxical association remains uncertain, including for CVD patients, and has been discussed in numerous studies; yet, the BMI is influenced by various components of the body composition. In regard to the lean mass, there are age-related, significant changes in the musculoskeletal system, with an annual muscle mass reduction of 2% after the age of 50 years [24]. Older individuals show a decrease in physical activity; nevertheless, training interventions produce significant improvements in physical function and hemodynamic parameters [25,26].

In this study, we firstly hypothesize that arterial stiffness correlates with body mass; secondly, the underlying mechanism of the association is the individual body composition of each patient. It is important to investigate whether body composition, in particular lean mass as a measure for muscle mass, is associated with arterial stiffness, making it a risk factor that could be influenced by lifestyle modifications for CVD prevention in patients with LEAD.

## 2. Materials and Methods

The present study was performed as a single-center, cross-sectional study. The inclusion criteria were met when patients were admitted to the outpatient department at the Division of Angiology, Medical University of Graz, due to current or previously diagnosed LEAD (Rutherford Classification 2–4), documented luminal stenosis >70% on ultrasound or angiography, or a history of endovascular or surgical revascularization. The exclusion criteria were defined as LEAD Rutherford 5 and 6 (tissue damage or loss), life expectancy <6 months, unstable coronary artery disease (CAD) or unstable cerebrovascular disease, uncontrolled diabetes, pregnancy, and an age of <18 years. Written informed consent was obtained before carrying out study-related procedures from all subjects who participated in the study.

Ethical approval for this study was given by the Ethics Committee, Medical University of Graz, Austria (EK Number 24–456 ex 11/12).

A general history was taken, and demographic data, anthropometric data, data on CVD risk factors, medication, and pertinent vascular examination records were collected. The ankle brachial index (ABI) was measured according to the current standard [27], and symptomatic LEAD was defined as an ABI of <0.9 and intermittent claudication, or a history of endovascular or surgical revascularization.

As a direct measure of arterial stiffness, the cfPWV and AIx were measured using the Vascular Explorer (Enverdis^®^, Dusseldorf, Germany). Individuals rested in the supine position for 15 min before measurements were taken. The fat mass was assessed using dual-energy x-ray absorptiometry (DXA) (iDXA, GE Lunar, Madison, WI, USA), and lean mass was calculated as weight (kg)–fat mass (kg). DXA has been found to be a reliable and valid method for measuring fat mass in the elderly, adults, and children [28,29]. All analyses were performed by two investigators. A standardized phantom was used daily to detect drifts in measurement and for the calibration of the equipment; regular equipment servicing was performed.

Continuous parameters were descriptively summarized using mean and standard deviation (SD) or median and interquartile range (Q1 and Q3, IQR) as appropriate; categorical data are presented as absolute and relative frequencies. Linear regression analysis was performed to assess the influence of lean body mass on arterial stiffness (the cfPWV and AIx) when accounting for the BMI. As patients included in our present study were the screening cohort for a randomized, controlled trial conducted by our study group and published previously [30], no sample size calculation was performed. The statistical analysis was performed in IBM SPSS Statistics version 26 (SPSS Inc., Chicago, IL, USA) and R version 3.6.1 (R Project, Vienna, Austria).

## 3. Results

A total of 412 patients were included in the study. Patients were predominantly men (72.09% vs. 27.91%) and the mean age (±SD) was 63.3 ± 10.1 years. 

**Figure 1:**
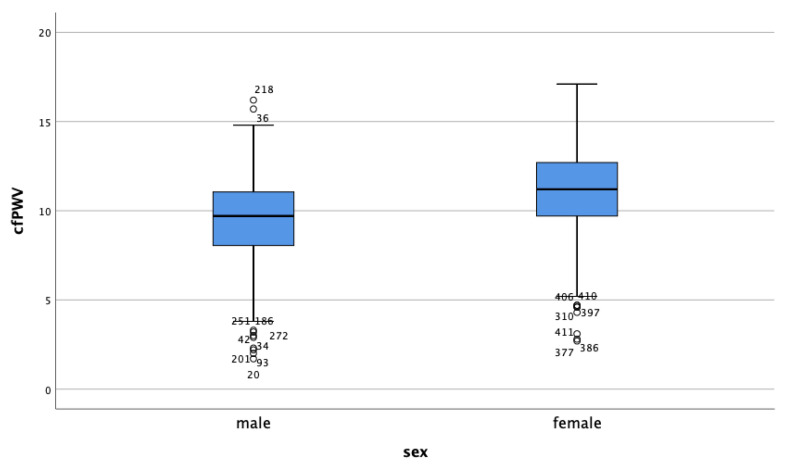
cfPWV according to sex.

**Figure 2:**
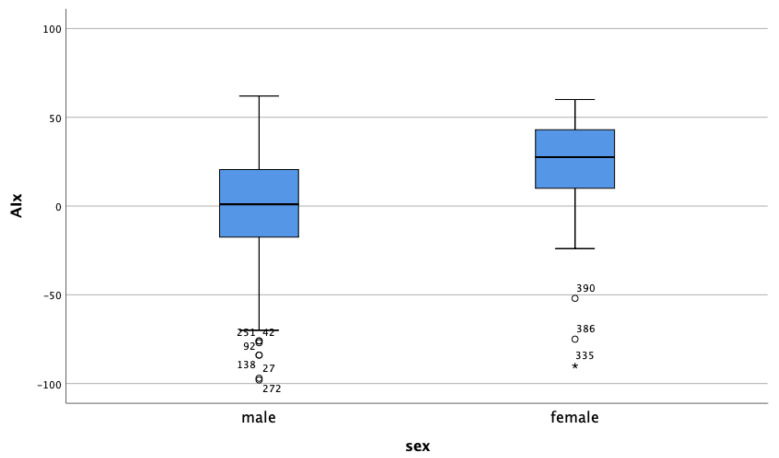
AIx according to sex.

The mean lean mass in the study population was 50.2 ± 9.4 kg, and lower for female patients compared to the male patients (39.6 ± 5.1 kg vs. 54.3 ± 7.3 kg). This contrasted with the fat mass percentage of 32.5 ± 6.4 in male patients and 41.2 ± 6.4 in female patients. The measurements of arterial stiffness, the AIx and cfPWV, were higher in the female study population compared to the male patients (Figure 1 and Figure 2).

The age of our study population also correlated positively with the AIx and cfPWV, as well as the percentage of fat mass, but negatively correlated with lean mass, as shown in Table 1.

Table 2 and Table 3 show the characteristics of the study patients, comorbidities, and values for the arterial stiffness parameters and lean mass in their BMI categories, for male and female patients, respectively. Of our study population, 81.3% had active or status post nicotine abuse (91.2% men; 62.6% women). Diabetes was present in 27% of our patients (26.9% men; 29.6% women), and hyperlipidemia in 93.1% (95.3% men; 95.7% women). Known hypertension was found in 78.7% of our patients (79.8% men; 82.6% women). Of our patient population, 10.4% (10.1% men; 12.2% women) already had a stroke, and known coronary artery disease (CAD) was found in 21.8% (24.2% men; 17.4% women). The BMI categories were determined in accordance with World Health Organization (WHO) definitions, with the BMI categories comprising underweight, normal, overweight, and obese. The majority of the male patients (71.04%) were in the overweight BMI category, whereas for the female patients, this figure was 57.39%. Only a handful of patients in this study fell into the obese (3.37% men vs. 6.09% women) and underweight (0.34% men vs. 1.74% women) categories.

In the regression analysis, we found that the AIx was significantly and negatively associated with lean mass (−0.28, 95% CI −0.48–0.09, *p* = 0.004) in male patients, even after accounting for the BMI. However, the cfPWV for male patients, as well as the Alx and cfPWV for female patients, was not significantly influenced by lean mass (Table 4).

## 4. Discussion

To the best of our knowledge, our present study is the first to assess the relationship between body composition and arterial stiffness in patients with LEAD. For patients with LEAD, our data clearly show that in overweight patients, lower lean mass is associated with increased arterial stiffness as measured by the AIx. This finding is consistent with data from Anoop et al., who showed that low, fat-free mass in Asian Indians with type 2 diabetes mellitus is also associated with greater arterial stiffness [31]. In previous studies, it was shown that the AIx has predictive value for all-cause mortality in patients with end-stage renal disease [7,32], for patients undergoing percutaneous coronary intervention (PCI) for CV events [33], as well as for CV events in patients with hypertension [34]. Furthermore, a meta-analysis conducted by Rodriguez et al. showed a consistent trend, considering the studies included, for measures of muscle tissue to be negatively related to arterial stiffness in unadjusted and adjusted analyses [35].

In young and healthy patients, it was found that peripheral lean mass may positively influence cardiovascular health [36], and central fat mass may increase arterial stiffness [37]. Although in other patient groups, as in the general population, the predictive value for this parameter is scarce [38], our data show that the AIx may be a suitable parameter for predicting CV events in patients with peripheral arterial disease.

Current guidelines suggest lifestyle modifications for patients with LEAD to reduce the risk of CVD [39,40]. If these lifestyle modifications include weight loss interventions, this may not be beneficial, as weight loss could also result in a reduction in muscle tissue [41].

Kingwell et al. demonstrated that a lack of physical activity is associated with increased arterial stiffness [42]. However, it should be noted that structured walking exercise alone, as suggested for patients with LEAD [39,40], has little or no effect on muscle loss prevention, while clinically meaningful gains in muscle mass can be achieved by progressive resistance training [41]. In a study conducted by Vun et al., a loss of lean mass of the thighs of the symptomatic as well as asymptomatic leg, after the completion of a standard, treadmill-based, supervised exercise in LEAD patients, was observed [43]. This finding is consistent with the results found in a randomized controlled study where patients lost strength in both limbs after changing from strength to treadmill training [44]. These findings suggest that treadmill-based exercise in patients with LEAD alone may not be beneficial for the gain of lean mass, through which the arterial stiffness measures could be influenced, as we found in our study. Still, walking for 2 h per week was found to be associated with a lower mortality in patients with diabetes [45]. Cardio-respiratory fitness and pain-free total walk capacity are improved by exercise training in patients with LEAD, as found by Parmenter et al. [46]; yet, aquatic walking decreases arterial stiffness and increases cardio-respiratory fitness in patients with LEAD [47]. Furthermore, it was found that in older individuals, training interventions improved the physical function and hemodynamic parameters significantly [25,26]. A combination of resistance and aerobic training was found to be more effective than either form of training alone in the prevention of adverse effects to the cardiovascular and musculoskeletal systems in an older population [25]. Moreover, it is necessary for older adults to undergo supervised physical activity for the maintenance of their physical function [48]. Several investigations found that a higher-intensity resistance component in the training resulted in a reduction in the blood pressure, and that greater muscle strength has an inverse association with the blood pressure, especially in older individuals [49,50,51,52]. Regular exercise is beneficial for older individuals to prevent CV events; age should not be a contraindication for exercise [53,54].

In contrast, Balkestein et al. stated that dietary changes, including weight loss, lower blood pressure, thereby achieving a reduction in arterial stiffness [55]. A combination of dietary changes and exercise reduces the loss of muscle tissue compared to dietary changes alone [56,57], and multimodal programs, including caloric restriction and progressive resistance training, may be beneficial for the minimization of muscle tissue loss and a reduction in arterial stiffness [41].

Changes in the cardiovascular system are most influenced by age and are predictors of adverse CVD events. The focal point of these changes is the vascular stiffness, which can be measured very precisely with the cfPWV [58]. In our present study, we also found that in patients with LEAD, age is significantly associated with greater arterial stiffness, and negatively associated with the lean mass. 

However, in our study, the cfPWV as a measure of arterial stiffness did not significantly correlate with lean mass in our study population. As our study predominantly included male patients, no statistical significance was found for either parameter of the arterial stiffness in female patients. Interestingly, Tanaka et al., in women with high levels of physical activity, observed no significant age-related increase in arterial stiffness [59]. As we did not examine the level of physical activity in our study population, we cannot exclude that this effect could account for the results we found for female patients. The small number of female patients in our study is one limitation that should be mentioned. Patients with LEAD are predominantly male, yet we cannot exclude that using a larger sample size of female patients would change the results. A further limitation of our study is the cross-sectional design; therefore, it remains unclear which mechanism is underlying and which lower lean mass contributes to a higher arterial stiffness. The strength of our study is the relatively large sample size of patients with LEAD, and the use of state-of-the-art methods for assessing the arterial stiffness and the body composition. 

Furthermore, the currently available data are not sufficient to prove a correlation between arterial stiffness and a reduction in CV events under treatment. Further randomized, controlled studies are needed to determine if a reduction in the cfPWV is also associated with a concomitant reduction in CV events. These data would be needed to demonstrate whether a therapeutic strategy to normalize arterial stiffness proves to be more effective in preventing CV events than standard care, or has a beneficial effect when combined with standard care [38]. However, vascular stiffness is an index of vascular health and may be influenced by an increased understanding of specific underlying mechanisms [60]. Additionally, further studies are needed to investigate the impact of exercise training in patients with LEAD regarding a reduction in arterial stiffness, gain of lean mass, prevention of CV events, and adverse limb outcomes.

## 5. Conclusions

For patients with peripheral arterial disease, our data show that lower lean mass is associated with increased arterial stiffness, as measured by the AIx, in overweight patients. The obesity paradox, as seen for patients with CVD, may be explainable by the higher lean mass in overweight patients with LEAD. Consequently, progressive resistance training and the gain of lean mass may be beneficial for a reduction in arterial stiffness in secondary prevention. Further studies are needed to show whether a reduction in arterial stiffness in LEAD patients is also associated with a concomitant reduction in adverse limb events.

## Figures and Tables

**Table 1:** Correlation between age, body composition, and arterial stiffness.

Variable 1	Variable 2	Correlation Coefficient	*p*-Value
Age	lean mass	−0.24	<0.05
Age	%-fat	0.16	<0.05
Age	AIx	0.26	<0.05
Age	cfPWV	0.21	<0.05

**Table 2:** Patient characteristics.

Characteristics	Total Cohort (*n* = 412)	Male Patients (*n* = 297)	Female Patients (*n* = 115)
Age (years)	63.3 ± 10.1	62.2 ± 9.5	66.3 ± 10.9
BMI	27.3 ± 4	27.4 ± 3.7	27.1 ± 4.8
Lean mass (kg)	50.2 ± 9.4	54.3 ± 7.3	39.6 ± 5.1
Nicotine abuse *n*	343 (81.3%)	271 (91.2%)	72 (62.6%)
Diabetes *n*	114 (27%)	80 (26.9%)	34 (29.6%)
Hyperlipidemia *n*	393 (93.1%)	283 (95.3%)	110 (95.7%)
Hypertension *n*	332 (78.8%)	237 (79.8%)	95 (82.6%)
Stroke *n*	44 (10.4%)	30 (10.1%)	14 (12.2%)
CAD *n*	92 (21.8%)	72 (24.4%)	20 (17.4%)
BMI category			
Underweight *n*	3 (0.7%)	1 (0.3%)	2 (1.7%)
Normal *n*	115 (27.9%)	75 (25.3%)	40 (34.8%)
Overweight *n*	277 (67.2%)	211 (71.0%)	66 (57.4%)
Obese *n*	17 (4.1%)	10 (3.4%)	7 (6.1%)

Mean (± SD); median (IQR); *n* (%).

**Table 3:** Patient characteristics II.

BMI Category		Male Patients	Female Patients
Underweight	*n* (%)	1 (0.3%)	2 (1.7%)
	AIx (mean, ±SD)	26	29.0 (±8.49)
	cfPWV (mean, ±SD)	8.9	12.65 (±2.05)
	Lean mass (mean, ±SD)	40.4	35.5 (±0.02)
Normal weight	*n* (%)	75 (25.3%)	40 (34.8%)
	AIx (mean, ±SD)	24.99 (±11.6)	30.79 (±14.56)
	cfPWV (mean, ±SD)	9.59 (±2.72)	10.18 (±3.24)
	Lean mass (mean, ±SD)	48.48 (±5.14)	36.23 (±3.81)
Overweight	*n* (%)	211 (71%)	66 (57.4%)
	AIx (mean, ±SD)	23.94 (±10.28)	31.14 (±8.93)
	cfPWV (mean, ±SD)	9.41 (±2.46)	11.35 (±3.04)
	Lean mass (mean, ±SD)	56.07 (±6.59)	41.33 (±4.35)
Obese	*n* (%)	10 (3.4%)	7 (6.1%)
	AIx (mean, ±SD)	20.4 (±20.4)	26.5 (±11.54)
	cfPWV (mean, ±SD)	9.22 (±1.95)	10.63 (±3.39)
	Lean mass (mean, ±SD)	63.97 (±6.02)	44.89 (±6.45)

**Table 4:** Regression analysis for arterial stiffness and lean mass according to sex.

	Sex	Variable	B Coefficient (95% CI)	*p*-Value
AIx	male	Lean mass BMI	−0.28 (−0.48–0.09) 1.14 (−2.04–4.33)	0.004 0.480
AIx	female	Lean mass BMI	−0.41 (−0.92–0.11) 2.12 (−3.11–7.35)	0.118 0.423
cfPWV	male	Lean mass BMI	−0.01 (−0.06–0.03) −0.10 (−0.89–0.68)	0.611 0.792
cfPWV	female	Lean mass BMI	−0.02 (−0.16–0.12) 1.09 (−0.38–2.56)	0.762 0.144

## Data Availability

The data presented in this study are available on request from the corresponding author.
